# *Dnd* knockout ablates germ cells and demonstrates germ cell independent sex differentiation in Atlantic salmon

**DOI:** 10.1038/srep21284

**Published:** 2016-02-18

**Authors:** Anna Wargelius, Sven Leininger, Kai Ove Skaftnesmo, Lene Kleppe, Eva Andersson, Geir Lasse Taranger, Rüdiger W Schulz, Rolf B Edvardsen

**Affiliations:** 1 Institute of Marine Research, P.O. Box 1870, Nordnes, NO-5817 Bergen, Norway; 2 Utrecht University, Science Faculty, Department Biology, Padualaan 8, NL-3584 CH Utrecht, The Netherlands

## Abstract

Introgression of farmed salmon escapees into wild stocks is a major threat to the genetic integrity of wild populations. Using germ cell-free fish in aquaculture may mitigate this problem. Our study investigated whether it is possible to produce germ cell-free salmon in F0 by using CRISPR-Cas9 to knock out *dnd*, a factor required for germ cell survival in vertebrates. To avoid studying mosaic animals, sgRNA targeting *alb* was simultaneously used as a visual tracer since the phenotype of *alb* KO is complete loss of pigmentation. Induced mutations for the tracer (*alb*) and the target (*dnd*) genes were highly correlated and produced germ cell-less fish lacking pigmentation, underlining the suitability of *alb* KO to serve as tracer for targeted double allelic mutations in F0 animals in species with prohibitively long generation times. This is also the first report describing *dnd* knockout in any fish species. Analyzing gene expression and histology of *dnd* KO fish revealed that sex differentiation of the somatic compartment does not depend on the presence of germ cells. However, the organization of the ovarian somatic compartment seems compromised in mutant fish.

Escaped Atlantic salmon (*Salmo salar* L.) are a major problem of fish farming as cultured fish are kept in open sea cages during ongrowth^1^. Use of sterile fish in salmon aquaculture could solve this problem, since it will prevent gene flow of domesticated salmon strains into wild populations^2^. Sterility in fish can be achieved experimentally by inactivating mRNAs essential for primordial germ cell (PGC) formation such as *dead end* (*dnd*), as shown in zebrafish, medaka, loach, goldfish and sturgeon^3^,^4^,^5^,^6^,^7^. In vertebrates, Dnd binds to 3′-UTRs of germ cell-specific RNAs, thereby protecting them against microRNA mediated degradation so that these RNAs can contribute to maintaining the PGC fate^8^. Also in mouse, loss of the *dnd* gene leads to germ cell-free sterile gonads^9^. However, in mammals, zygotic transcription replaces maternal RNA already at the 1–2 cell stage while in fish this occurs later at the mid-blastula transition^10^. This feature is relevant regarding the function of some maternal RNAs to maintain germ cells, such as *piwil1, piwil2* and *vasa*, which are sufficient to initiate germ cell formation in homozygous mutant fish for these genes, although germ cell loss then occurs at later stages^11^,^12^,^13^. However, previous studies in fish have only assayed the function of *dnd* when both maternal and zygotic *dnd* mRNA is lost during early development^3^,^4^,^7^. It remains therefore to be elucidated if maternal *dnd* mRNA can rescue the survival of germ cells in a *dnd* KO fish. Loss of germ cells in fish also affects the somatic sex of the gonad differently since loss of PGCs leads to an all-male phenotype in medaka, zebrafish and tilapia while both males and females develop in germ cell-free loach and goldfish^3^,^4^,^5^,^14^,^15^. In mammals (mouse), knock out of the *dnd* gene leads to an all-male offspring^9^. Whether or not the absence of germ cells is relevant for sex differentiation is not known in salmonids.

Recently, the CRISPR-Cas9 system for targeted mutagenesis has been implemented in zebrafish using a codon-optimized Cas9^16^. This technology has the benefit of efficiently inducing double allelic mutations already in F0 in zebrafish, tilapia, Atlantic salmon and killifish^15^,^17^,^18^,^19^. The CRISPR-Cas9 system is therefore suited to develop new sterility models in Atlantic salmon by a germ cell-specific candidate gene approach. This requires the identification of genes without functionally redundant paralogs that could obscure the loss of function phenotype. Although, the partially tetraploid genome of salmonids complicates the search for genes without paralogs^20^,^21^, *dnd* is a suitable candidate gene as there are no known paralogs in Atlantic salmon. In the past we have shown that CRISPR-Cas9 can be used to knockout pigmentation genes in salmon^18^. Targeting the *slc45a2* (*alb*) pigmentation gene resulted in a range of mosaic phenotypes from the lack of pigmentation in a few cells to completely albino animals. This graded mosaicism is a general problem in F0 KO animals. However, working with F0 animals is often the only feasible approach for functional studies in non model species with a long generation time like the Atlantic salmon.

In the present study we carried out double and single KO experiments in Atlantic salmon using CRISPR/Cas9, targeting *dnd* or both *dnd* and *slc45a2* (*albino/alb*). Since CRISPR-Cas9 RNAs targeting *alb* produce a complete albino phenotype when the mutation is double allelic, we hypothesized that this feature could indicate double allelic gene loss following the injection of a second, unrelated CRISPR-RNA into the same embryo. Hence loss of pigmentation in double injected embryos could also indicate a high mutation degree of the second targeted gene. Here we show that albino *dnd/alb* KO fish were germ cell free in F0. Hence, we could describe for the first time in fish that CRISPR-Cas9-mediated KO of *dnd* leads to complete loss of germ cells in the F0 generation, also demonstrating that maternal RNA cannot compensate for the zygotic loss of the gene. Finally, we found that germ cells are not required for female sex differentiation in Atlantic salmon but possibly for establishing a normal structure of the somatic compartment in the female gonad.

## Results and Discussion

Targeted mutagenesis in species with long generation times (3 years in Atlantic salmon) is challenging because it takes a long time to produce F2 individuals of a single double allelic mutation. The recently developed CRISPR-Cas9 system efficiently generates double allelic mutations in the F0 generation in many species, such as *Xenopus*, zebrafish, tilapia and Atlantic salmon, thereby allowing functional characterization after a comparatively short period^15^,^17^,^18^,^22^. In loss of function experiments it is essential that individuals do no longer carry wild type versions of the gene of interest. As we have previously observed, the degree of loss of function can be monitored in studies on the pigmentation genes *alb* and *tyr*^18^, where CRISPR-Cas9-mediated KO of *alb* or *tyr* produced fish with different degrees of mosaicisms. This feature is a general problem when studying gene function in F0: It is not known to what extent wildtype sequences may rescue the phenotype produced by (incomplete) mutagenesis. In CRISPR-*alb* individuals many fish completely lacked pigmentation and fin clip analyses confirmed the absence of wild type variants in those, analyzing about 100 clones from PCR products^18^. This result suggested a 100% KO in these F0 fish. In a similar way, *tyr* KO also causes loss of pigmentation in F0 but unfortunately, this phenotype is not maintained in juvenile and adult fish (data not shown), making *tyr* an unsuitable tracer for phenotypic identification of mutants. Based on our previous studies, we hypothesized that CRISPR-Cas9-induced double KO of *alb* and *dnd* would exhibit similar efficiencies for both genes. Especially since both genes induced mutations (in whole embryo assays) in 40% of the injected embryos (4 of 10 embryos for each targeted gene). Double and multiple gene knockouts using CRISPR-Cas9 have previously been reported for zebrafish and mouse models^23^,^24^. PCR screening and sequencing of embryos approximately 14 days after injection confirmed successful gene editing by CRISPR-Cas9-induced KO in both *alb* and *dnd* (Supplementary Figure S1). Further PCR screening for *dnd* of 271 fin clips from juvenile fish resulted in 17% (n = 45) fish that carried indel mutations in *dnd* (Table 1), although only 28 of them displayed an albino phenotype (complete or mosaic). However, all the fish displaying an albino phenotype also carried mutations in the *dnd* gene (Table 1, Supplementary Figure S1) and 98% of all *dnd* KO fish (44/45) also carried mutations in the albino gene. This shows that CRISPR-Cas9-mediated *alb* KO can be used as a visual tracer for gene editing in salmon, facilitating the phenotypic analysis of mutations in genes with unknown functions. We also attempted single gene mutations for *dnd* (without *alb*) where 28% (63/222) of injected fish carried indels.

**Figure 1:**
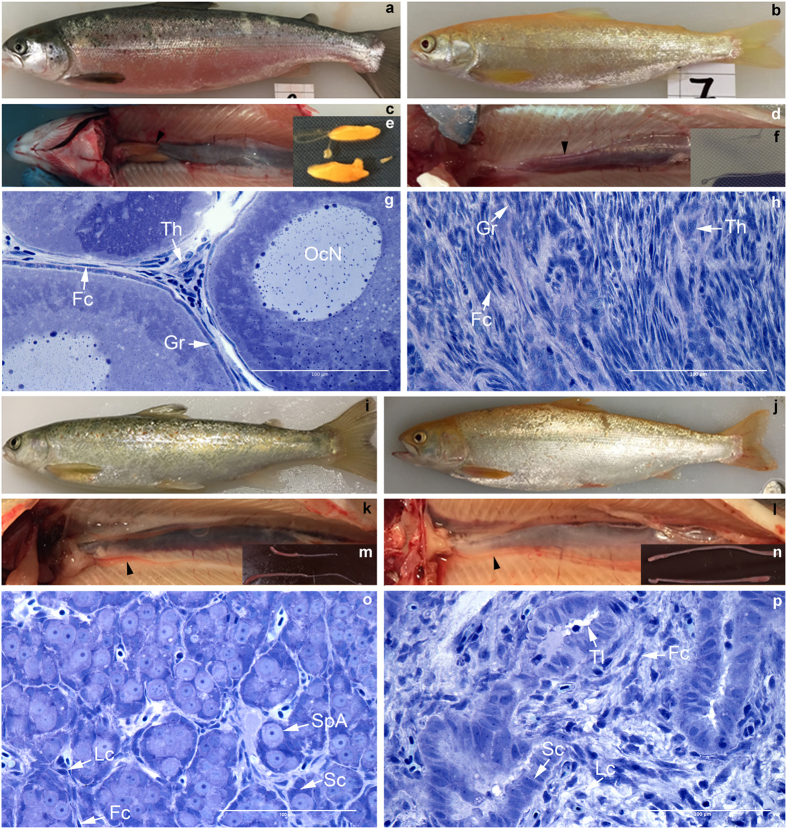
Gross morphology and histology of one year old *dnd*/*alb* KO and control fish. The right panel shows *dnd*/*alb* KO fish (**b**,**d**,**f**,**h**,**j**,**l**,**n**,**p**). The left panel shows control fish (**a**,**c**,**e**,**g**,**i**,**k**,**m**,**o**). Fish b is a female *dnd*/*alb* KO fish, d and f: gross morphology of the female *dnd*/*alb* KO in comparison to the gross morphology of the control female (**a**,**c**,**e**,**f**): lack of the ovarian bulb in comparison to control (**e**). G and h: histology of the female gonad in *dnd*/*alb* KO (**h**) in comparison to control ovary (**g**). Fish j is a male *dnd*/*alb* KO fish, l and n: gross morphology of the male *dnd*/*alb* KO in comparison to the gross morphology of the control male (**i**, **k**,**m**,**o**,**p**): histology of the male gonad in *dnd*/*alb* KO (**p**) in comparison to control testis (**o**). Scale bar in (**g**,**h**,**o)** and p 100 μM. Arrowheads in (**c**,**d**,**k**,**l**)indicate gonads. Abbreviations: OcN – oocyte nucleus; Gr – granulosa cell; Th – theca cell; Fc – fibrocyte; Lc - Leydig cell; Sc - Sertoli cell; SpA - spermatogonium A; Tl - tubular lumen. Supplementary Table S2 lists the length, weight, age and sampling date of individual fish.

In this study, we analyzed gonads of 30 fish in total, of which 24 had prior to sampling been determined to contain *dnd* mutations in finclips. In the 30 fish sampled, 10 were *dnd*/*alb* KO with complete albino phenotype, 14 were gene-edited for *dnd* only, and 6 control fish (3 of each sex, Supplementary Table S2 and Supplementary Figure S2). We selected six female and four male *dnd*/*alb* mutated fish between 12–15 months of age with complete albino phenotypes and *dnd* mutations in fin clips^18^ for visual and histological examinations. Hence, we supposed they also had biallelic mutations for *dnd* (Fig. 1, Supplementary Table 2, fish 4–9 and 17–20, Supplementary Figure S2). Fish were visually examined, opened and all organs except the gonads were removed from the body cavity. Control females at the age of 12–18 months typically show two yellow-orange colored ovarian bulbs in the cranial part of the body cavity (Fig. 1c,e). At this stage the ovaries contain many previtellogenic oocytes at the perinucleolar stage of development (Fig. 1g). The transition into the secondary growth phase has not started yet, i.e. cortical alveoli are absent. In *dnd*/*alb* KO females ovarian tissue showed a very different appearance (Fig. 1b, Supplementary Table S2, fish 4–9, Supplementary Figure S2, fish 7–8 and 10). The yellow-orange ovarian bulbs were missing at the cranial end of the ovaries that instead was a thin, whitish thread of tissue over its complete length (Fig. 1d,f), similar to the caudal part of the control ovary (Fig. 1c,e). Histological analysis of the cranial part of mutant ovaries showed that neither primary oocytes (Fig. 1h, Supplementary Figure S2, fish 7–8 and 10) nor pre-meiotic oogonial cells were present. Only fibrocytes, extracellular connective tissue elements and blood vessels were found, as well as cells that may represent granulosa and theca cells (Fig. 1h, Supplementary Figure S2, fish 7–8 and 10); future work will have to verify the identity of the different cell types found in the germ cell-free ovary. These observations suggest that germ cell-free female gonads develop an ovarian somatic structure resembling the situation found in loach and goldfish^4^,^5^, distinct from zebrafish, medaka and tilapia where all germ cell free fish develop somatic testes^4^,^5^,^15^. Macroscopic inspection of gonads in control males (Fig. 1k,m) showed the typical, thread-like appearance of immature testes. Histological analysis revealed prepubertal testis tissue with spermatogenic tubules containing numerous type A spermatogonia enveloped by Sertoli cells, and interstitial tissue with blood vessels, Leydig cells, and connective tissue elements (Fig. 1o). Macroscopic inspection of the four *dnd/alb*-KO male fish upon dissection (Fig. 1l,n, Supplementary Figure S2, fish 18, 20 and 28) revealed an immature thread-like testis (Fig. 1l,n, Supplementary Figure S2, fish 18, 20 and 28), not unlike the situation in control males (Fig. 1i,k & m, Supplementary Table S2). However, histological analysis of the mutant testes showed that germ cells were absent and that the germ cell-free “spermatogenic tubuli” contained Sertoli cells only. The interstitial area of the mutant testes appeared to be normal, showing for example connective tissue elements, blood vessels and Leydig cells (Fig. 1p). The results from the male *dnd*/*alb* KO fish is similar to what has been observed in *dnd* knock down studies in zebrafish, medaka, loach, goldfish and tilapia: the germ cell-free male gonad seems to develop as a somatic testis^3^,^4^,^5^,^14^,^15^. Our morphological results suggest that salmon show a similar sex differentiation system as loach and goldfish, while in medaka, zebrafish and tilapia germ cells are required for the female differentiation of the somatic gonad^3^,^4^,^5^,^14^,^15^. In mammals (mouse) germ cell-less *dnd* knock out animals are all males^9^.

Interestingly the germ cell-free ovary showed an unorganized appearance (Fig. 1h, Supplementary Figure S2 fish 7–8 and 10), while in the germ cell-free testis, the interstitial tissue was clearly separated from (germ cell-free) spermatogenic tubules that contained a proliferating Sertoli cell epithelium and formed a more organized structure that macroscopically was similar to a normal testis (Fig. 1l,p, Supplementary Figure S2 fish 18, 20 and 28). This suggests that establishing the testis tissue architecture and its main components, the spermatogenic tubules and the interstitial compartment, is not depending on the presence of germ cells while the germ cell-free somatic component of the ovary remain less organized in the salmon. This difference between female and male fish was not observed in loach and goldfish although they also retained the female state of the gonad in spite of lack of germ cells^4^,^5^. This may be related to the fact that we have kept the fish for a longer time and thereby have been able to observe differentiation of the gonads in germ cell free salmon. Studies in sterile W/Wv mouse lacking germ cells showed that lack of follicle formation lead to development of thin ovaries^25^. This study supports our observation in salmon since we also observe a thin streak of ovary and lack of follicles in germ cell free female gonads.

We also sampled gonads from four randomly selected females and ten randomly selected males of *dnd* mutated fish (i.e. no attempt of *alb* KO). These fish could have a mosaic mutation pattern and only some tissues would be depleted of functional *dnd*. Among those, one of the females had gonads containing germ cells, indicating the absence of *dnd* mutations in cells responsible for normal germ cell development (Supplementary Table S2, fish no 13). In the remaining 3 females, the ovarian bulb was missing (Supplementary Table S2, fish no 10–12, Supplementary Figure S2 fish 10) and histological analysis showed that three of them lacked germ cells (Supplementary Table S2 fish no 10–11, Supplementary Figure S2, fish no 10). The histology sample from female number 12 was lost but since the sample for gene expression analysis showed *cyp19a1* but not *vasa* expression we assume that this female also lacked germ cells.

From the ten male *dnd* mutated fish, histological analysis showed that five displayed a complete lack of germ cells (Supplementary Table S2, Supplementary Figure S2, fish no 21–22, 28–30). Another male had an immature gonad with germ cells (Supplementary Table S2, fish no 23). Four males were maturing showing all stages of testis development but the testicular anatomy was atypical since only half of the length of the gonad contained all germ cell stages during maturation (Supplementary Table S2, fish no 24–27 and Supplementary Figure S3 fish no 25 and 27). While a mature control testis (S2, control) contained spermatogenic tubuli filled with sperm that were lined by an epithelium consisting of type A spermatogonia enveloped by Sertoli cells, the mosaic testis of male #25 showed in the thin, cranial part a histology similar to a *dnd* mutant male, except for the presence of spermatozoa. However, all other germ cell stages were missing and the testis tubuli were lined by a Sertoli cell epithelium, as seen in the mutant testes. Analyzing the thick area of the mosaic testis of male #27 showed that from type A spermatogonia to spermatids, all germ cell stages were present and that the lumina were filled with spermatozoa. Since the lumina of the spermatogenic tubules are anastomosing in fish, we assume that the spermatozoa found in the thin areas are derived from spermatogenic tissue in the thick areas of mosaic testes. These results seem to indicate that in case of a mosaic mutation, only a few germ cells had reached the testis, which thus remained incompletely populated by germ cells. Only 57% of the *dnd-*mutated fish displayed a complete loss of function phenotype while 100% of the *dnd/alb* KO fish displayed loss of function (table1). For the *dnd/alb* KO analysis we selected only individuals completely lacking pigmentation since these were more likely to have a double allelic mutation for the *alb* and thereby also for the *dnd*. This demonstrates the benefit of using *alb* KO as a tracer for double allelic mutants in functional studies of candidate genes in Atlantic salmon.

**Figure 2:**
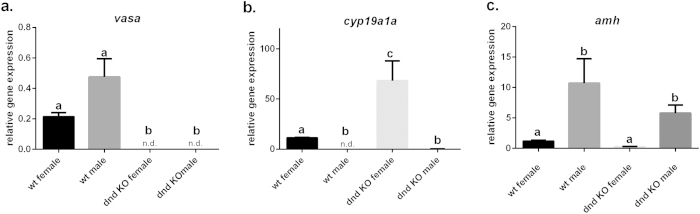
Gene expression of *vasa*, *cyp19a1a* (*p450 aromatase*) and *amh* (*anti müllerian hormone*) in gonads of male and female *dnd* KO (n = 4 per sex) and control fish (n = 3 per sex). Gonads were obtained from 12–18 months old fish. Individual fish used for qPCR are indicated in Supplementary Table S2. Samples assayed are listed on the x-axis, while the y-axis indicates the relative abundance of the transcripts in relation to the normalization factor *elongation factor1α*. Data are presented as ± SEM. Significant gene expression differences between groups are indicated by letters (**a**–**c**); n.d. - not detected.

We used the germ cell marker *vasa* to confirm absence of these cells in gonads of *dnd* KO fish by qPCR analyses of whole gonad preparations^26^,^27^. To confirm gonad specific expression of *vasa*, six other tissues in proximity of the gonads were assayed for *vasa* expression. Results confirmed tissue specificity of *vasa* in gonads (Supplementary Figure S4a). All fish devoid of germ cells upon histological examination also lacked *vasa* expression in the complete gonad, confirming the sterility of these fish (Fig. 2a). To confirm the loss of function, we quantified *dnd* transcript levels in whole gonad extracts. Similar to *vasa*, no expression was found in gonads of *dnd* KO fish (Supplemental Figure S5a). Fish used for qPCR analyses are listed in Supplementary Table S2. Several genes have been used to phenotypically describe the sex of germ cell-free gonads. In zebrafish *cyp19a1a* (*p450 aromatase*) catalyzes the conversion of testosterone into estrogen and is highly expressed in granulosa cells of the ovary and is thereby a reliable marker of somatic sex of the ovary^28^. We could not detect *cyp19a1a* in 7 other tissues close to the gonads nor in testis tissue (Supplementary Figure S4b). Also, studies in loach and goldfish used female *cyp19a1a* overexpression to confirm that the germ cell-free gonad had developed into a somatic ovary^4^,^5^. In contrast, zebrafish, medaka and tilapia with germ cell-free gonads all developed phenotypic male gonads and lacked *cyp19a1a* expression^3^,^7^,^15^,^29^. Our data showed that genetically female, germ cell-free salmon gonads overexpressed *cyp19a1a* in comparison to genetically male gonadal tissue lacking noticeable expression of this gene (Fig. 2b). In the female *dnd* KO fish *cyp19a1a* was higher compared to control females, which may reflect a relative over-representation of somatic genes in the germ cell-free ovary. A second female marker, the *foxl2*, a transcription factor involved in ovarian development and growth, supports this result showing a similar pattern as *cyp19a1a* in assayed gonads^30^(Supplementary Figure S5b).

A suitable marker to phenotypically characterize the immature male gonad seems to be *anti müllerian hormone* (*amh*). This gene is highly expressed in the immature testis prior to the onset of gonad maturation^31^, and work in zebrafish has shown that there is a clear male-biased overexpression at early stages of gonadal development^32^. Our results show that *amh* is highly expressed in immature wild type testes and in the *dnd KO* male gonad, confirming the male phenotype of the germ cell-free gonad (Fig. 2c). Analysis of *amh* expression in 6 other tissues in salmon verified what has been reported in zebrafish^28^, namely tissue specific expression in the gonad and male-biased overexpression in the testis (Supplementary Figure S4c). Our morphological and molecular analyses clearly show that germ cells do not contribute to sex differentiation in salmon. The sex determination factor *sdY*^33^ is expressed in the male somatic gonad of Atlantic salmon (data not shown) which further strengthens the view that germ cells are not essential for sex differentiation in salmonid fishes. Another known male biased marker, the *sox9a gene*^28^, showed higher expression in males, both in control and germ cell less individuals compared to females (Supplementary Figure S5c). While other functional studies in fish reported the knockdown of *dnd* RNA using morpholinos^3^,^4^,^5^,^14^, we have knocked out the gene, as reported for studies in mouse^9^. The previous functional studies on *dnd* in fish used ATG-targeting morpholinos and did therefore not address the question if maternal *dnd* RNA plays a role in Dnd-mediated PGC formation, because these morpholinos targeted both zygotic and maternal RNA. Since zygotic gene expression is not turned on until the onset of gastrulation in fish, our study shows that zygotic *dnd* RNA is required for the migration of germ cells to the gonads^10^. A study in tilapia showed that CRISPR-Cas9 KO of the *nanos-3* gene resulted in germ cell free gonads, further strengthening the notion that maternally contributed factors cannot rescue germ cell development and survival^15^. Some of the questions that remain to be elucidated in future studies are when the lack of zygotic *dnd* results in germ cell death and if and how, compared to wild type siblings, somatic gonadal tissue embarks on subsequent developmental steps, such as sexual maturation.

There seem to be at least two manners of female sex differentiation of somatic gonadal cells in fish, one that depends on the presence of germ cells (e.g. zebrafish, medaka, tilapia), and one that does not (e.g. loach, goldfish, salmon). There is no obvious phylogenetic aspect in this grouping, considering that goldfish and zebrafish are closely related species. Recent work showed that zebrafish have lost a sex-determining region on chromosome 4 during domestication^34^ and it would be interesting to re-evaluate somatic gonadal sex differentiation in *dnd* knock down individuals of wild zebrafish strains. In domesticated zebrafish, the number of meiotic cells during the sex differentiation period is relevant for sustaining the female identity and an elevated *cyp19a1a* expression^35^. Also loss of a gene that still does allow PGC survival and their differentiation into gonial cells but not germ cell entry into meiosis (e.g. loss of *piwil1*^11^) led to an all-male phenotype in zebrafish. It is possible that in the absence of the sex-determining region a signal derived from a sufficiently high number of meiotic germ cells is required to support *cyp19a1a* expression and hence female sex differentiation of the somatic gonadal cells in zebrafish, and perhaps also in species like medaka and tilapia. In other species, like salmon *cyp19a1a* expression can be high in the absence of germ cells which can prevent formation of an all-male phenotype. In accordance with previous suggestions^36^, we therefore hypothesize that estrogen signaling is decisive for female sex differentiation and that the elevated expression of *cyp19a1a* required for this to occur can be achieved by germ cell dependent as well as germ cell independent mechanisms in different fish species.

Our study shows that the complete *alb* KO phenotype is a reliable tracer for the concomitant KO of genes involved in unrelated processes (100% accuracy in our study using *dnd*). Importantly, the biallelic KO allows functional studies in F0 of Atlantic salmon, and with high probability also in other species with a long life cycle which usually prohibits generating an F2 generation. We reveal that *dnd/alb* F0 mutants completely lacking pigmentation are also devoid of germ cells, suggesting that maternally transferred *dnd* mRNA cannot compensate for the zygotic loss of this gene. In addition, our experiments show that germ cells are not essential for gonadal sex differentiation in Atlantic salmon since the somatic sex was maintained in male and female germ cell free fish. Finally, we saw disorganized, very thin gonads only in germ cell free females while the somatic compartment in sterile males appeared normal, supporting the notion that germ cells exert an organizing effect on the somatic compartment in the salmon ovary but not in the testis.

## Experimental procedures

### Cloning and expression of CRISPR-Cas9 construct*s*

The *Salmo salar dnd* mRNA sequence (acc. JN712911^27^) was used to find the genome scaffold containing the *dnd* gene (jcf1000428558_0-0, assembly Ssa_ASM_3.6.fasta (Acc. No. AGKD00000000.3)). *Dnd* target site sequences and CRISPR oligos are listed in Table S1. Oligonucleotides for *slc45a2* (*alb*) are described in Edvardsen *et al.*^18^. The *dnd* target site was selected in the exon encoding the DNA-binding domain. To avoid off-target affects we searched the whole salmon genome (No. AGKD00000000.4) for binding sites for the oligos selected for the CRISPR target for both *dnd* and *alb*, no specific binding was identified for the targets used. Cloning of CRISPR target sequences and preparation of *cas9* mRNA and *in-vitro* transcription was conducted as previously described^18^ with the following exceptions: for *in-vitro* transcription of gRNA we used the HighScribe T7 High Yield RNA Synthesis Kit (NEB) according to the protocol for short transcripts and purified gRNA via an RNeasy column (Qiagen) using 3.5 vol. of 100% EtOH.

### Experimental setup and injections

Injection procedures were carried out as described previously^18^, using 50 ng/μl of each gRNA and 150 ng/μl of *cas9* mRNA. At 8–10 months after fertilization, when fish were around 10–50 g they were tagged intraperitoneally with 8 mm passive inductive transponder-tags (ID-100 A Microtransponder, Trovan Ltd) and the adipose fin was clipped as a source for DNA extraction. Visual inspection revealed the number of *alb* KO fish due to their albino phenotype^18^ and all pit-tagged fish were screened for *dnd* mutations in the fin-clip sample. Fish bearing *dnd* mutations but no visual albino phenotype were screened for *alb* mutations as previously published^18^. All *dnd* KO fish were screened for their sex using double exon *sdY* PCR^37^ to be able to select genetic females and males for histological studies. At 13–15 months of age, 6 control fish (3 of each sex) and 12 *dnd* KO fish (8 males and 6 females) were sedated and euthanized using 10 mg/l metomidate (Syndel, Canada). Fish were dissected and the whole length of one gonad was immediately frozen in liquid nitrogen for RNA extraction and qPCR analyses. The whole length of the other gonad was fixed in 4% glutaraldehyde for embedding in plastic (Technovit 7100).

### Screening for mutation*s*

To screen for mutations, 10 embryos from each injection batch were dechorionated and frozen for DNA extraction at the 17 somite stage at approx. 2 weeks of age. DNA extraction from embryos and screening for indel mutations was done as described in Edvardsen *et al.*^18^. DNA from fin-clips of pit tagged fish was extracted using the DNeasy 96 Blood & Tissue Kit (Qiagen). PCR primers for screening are listed in Supplementary Table S1. We did not clone PCR products to further investigate *dnd* indel variations because we expected a high degree of mosaicism in the F0 generation and no wildtype variants in full albino fish due to our previous analyses of *alb* (*slc45a2*) and *tyr* indel variations^18^.

### Histology of gonad*s*

For morphological analyses, the whole length of one gonad was fixed in 4% phosphate-buffered glutaraldehyde at 4 °C overnight. Subsequently, the tissue was dehydrated, embedded in Technovit 7100 (Electron Microscopy Sciences), sectioned at 4 μm thickness, and stained with toluidine blue, according to conventional histological procedures.

### qPCR

RNA was extracted from the whole length of one gonad using the RNeasy Kit (Qiagen) according to the manufacturer’s instructions. Purified RNA was then treated with the turbo DNAfree kit (Ambion) to remove trace amounts of contaminating DNA. The treated RNA served as input for reverse transcription reaction using the VILO cDNA synthesis kit (Invitrogen). 2 μl of a 1/20 dilution of the final cDNA was used in a 6 μl Fast Taqman qPCR reaction (Applied Biosystems) for *vasa*, *cyp19a1a* and *amh* Fast *Sybr* green was used (Applied Biosystems). Primers and probes for the amplicons are listed in Supplementary Table S1. All PCR reactions were run in duplicate with both no template and –RT controls. Using the standard-curve method, PCR efficiencies were verified to be approximately equal between target and the reference gene *elongation factor1α* (*ef1a*, using 100, 50, 25, 12.5, and 6.25 ng RNA). qPCR was performed on a SDS 7900HT Fast Real-Time PCR system (Applied Biosystems),with the following thermal cycling conditions: 95 °C for 20 sec, and 40 cycles of 95 °C for 1 sec followed by 60 °C for 20 sec. For screening of tissue specific gene expression we used cDNA prepared from tissue samples presented in Kleppe *et al.* 2015^21^.

### Statistical analysis

The statistical tests on qPCR results were performed using GraphPad Prism 5.04 (GraphPad Software Inc). A p-value of ≤0.05 indicates significant difference. Due to the low number of replicate qPCR samples (n = 3–4) none of the samples showed a normal distribution. Therefore, the non parametric Kolmogorov-Smirnov test was applied to calculate significant differences between samples.

### Use of experimental animals

All experiments herein have been approved by the Norwegian Animal Research Authority (NARA, permit number 5741). Welfare and use of these experimental animals was performed in strict accordance with the Norwegian Animal Welfare Act of 19^th^ of June 2009, in force from 1^st^ of January 2010.

## Additional Information

**How to cite this article**: Wargelius, A. *et al.* *Dnd* knockout ablates germ cells and demonstrates germ cell independent sex differentiation in Atlantic salmon. *Sci. Rep.* **6**, 21284; doi: 10.1038/srep21284 (2016).

## Supplementary Material

Supplementary Information

## Figures and Tables

**Table 1:** Percent mutated fish and clear phenotypes induced by CRISPR-Cas9.

targeted gene	*dnd*mutations	*dnd* mutations and albino phenotype	*dnd* and *alb* mutations	germ cell free fish
*dnd*	28% (63/222)	–	–	57% (8/14)
*dnd/alb*	17% (45/271)	100% (28/28)	98% (44/45)	100% (10/10)^1^

^1^All sampled fish with a complete albino phenotype.
