# Supplementary material for: Dnd knockout ablates germ cells and demonstrates germ cell independent sex differentiation in Atlantic salmon
Source: Sci Rep. 2016 Feb 18;6:21284. doi: 10.1038/srep21284 (PMC4758030; doi:10.1038/srep21284)
Supplement: Supplementary Information [file srep21284-s1.pdf]

Supplementary information for the article;

Dnd knockout ablates germ cells and demonstrates germ cell independent sex differentiation in Atlantic salmon

Anna Wargelius[1*], Sven Leininger[1, 2], Kai Ove Skaftnesmo[1], Lene Kleppe[1], Eva Andersson[1], Geir Lasse Taranger[1], Rüdiger W Schulz[1,3], Rolf B Edvardsen[1]

[1]Institute of Marine Research, P.O. Box 1870, Nordnes, NO-5817 Bergen, Norway
[2] present address: MSD Animal Health Innovation AS, Thormøhlensgate 55, 5008 Bergen, Norway

[3] Utrecht University, Science Faculty, Department Biology, Padualaan 8, NL-3584 CH Utrecht, The Netherlands.

Contact

*Corresponding author, anna.wargelius@imr.no

Supplementary material

Supplementary Figure S1. Representative analysis of CRISPR-Cas9-mediated knock out of *dnd* and *alb*: chromatograms showing sequencing results of amplicons of *dnd* and *alb* around the CRISPR site (light green arrows). A: fish with no mutations; B: a male and C: a female fish with mutations indicated by multiple peaks starting in the CRISPR site and proceeding downstream.

Supplementary Figure S2. Gross morphology and histology of six fish (n=3 of each sex) showing the germ cell free phenotype. Numbers on fish refer to the fish presented in Supplementary table S2, where more information on each fish is found (length, weight, age, sampling date and phenotype of sectioned gonad). (a) Picture of fish, (b) gross morphology of gonads and (c) section of respective gonad. Scale bar in 7c, 8c, 10c, 20c, 18c and 28c, 100 μM.

Supplementary Figure S3. Gross morphology and histology of one control fish and 2 mosaic dndKO fish, numbers on *dnd* KO fish refers to the fish presented in Supplementary table S2, (listing length, weight, age, sampling date and phenotype of sectioned gonad). (a) picture of fish, (b) gross morphology of gonads and (c) section of respective gonad. Scale bar in control histology, 25c and 27c, 100 μM.

Supplementary Figure S4. Relative gene expression of *vasa* (a), *cyp19a1a* (b) and *amh* (c) in adult Atlantic salmon tissues (n=3 per tissue; ovary, testis, liver, brain, head kidney, intestine, muscle and spleen). Samples assayed are listed on the x-axis, while the y-axis indicates the relative abundance of the transcripts in relation to the normalization factor *elongation factor1α*. For each gene all values were calibrated to the sample with the lowest ΔCt; therefore the highest gene expression value was set to 1. All data are shown as SEM. n=3.

Supplementary Figure S5. Relative gene expression of *dnd* (a), *foxl2* (b) and *sox9a* (c) in adult Atlantic salmon tissues in gonads of male and female *dnd* KO (n=4 per sex) and control fish (n=3 per sex). Gonads were obtained from 12-18 months old fish. Individual fish used for qPCR are indicated in Supplementary table S2. Samples assayed are listed on the x-axis, while the y-axis indicates the relative abundance of the transcripts in relation to the normalization factor *elongation factor1α*. Data are presented as ±SEM. Significant gene expression differences between groups are indicated by letters (a, b and c); *n.d.* - not detected.

Supplementary table S1. Oligonucleotides and primers used in the study

Supplementary table S2. Overview of all fish sampled and used in the analysis of mutated and control fish. The table shows fish number (No), sample type (Sample), sex (F/M), phenotype of gonad, if sample was in included in qPCR analysis, sampling date and length, weight and age (months) of fish.

Supplementary Figure S1

A control fish

alb

dnd

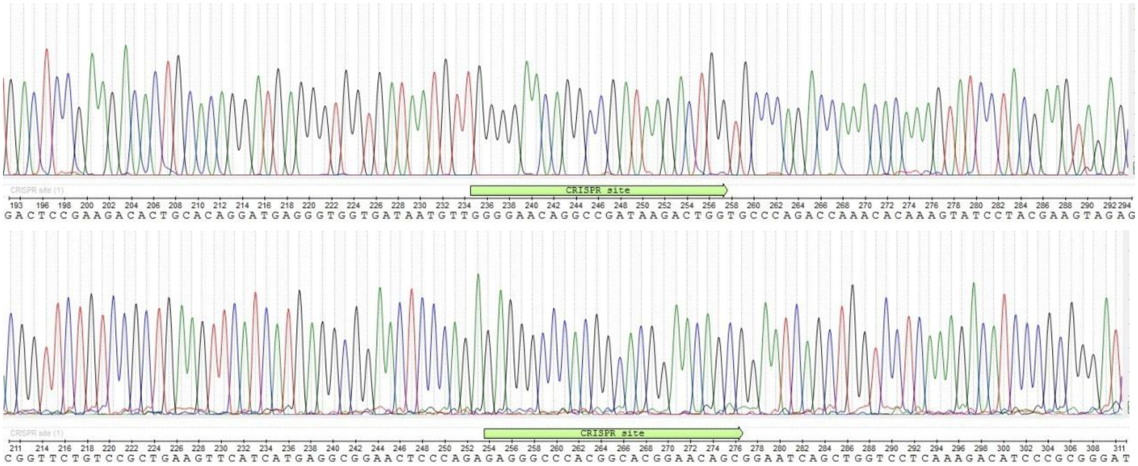

B *dnd/alb* KO fish, male

alb

dnd

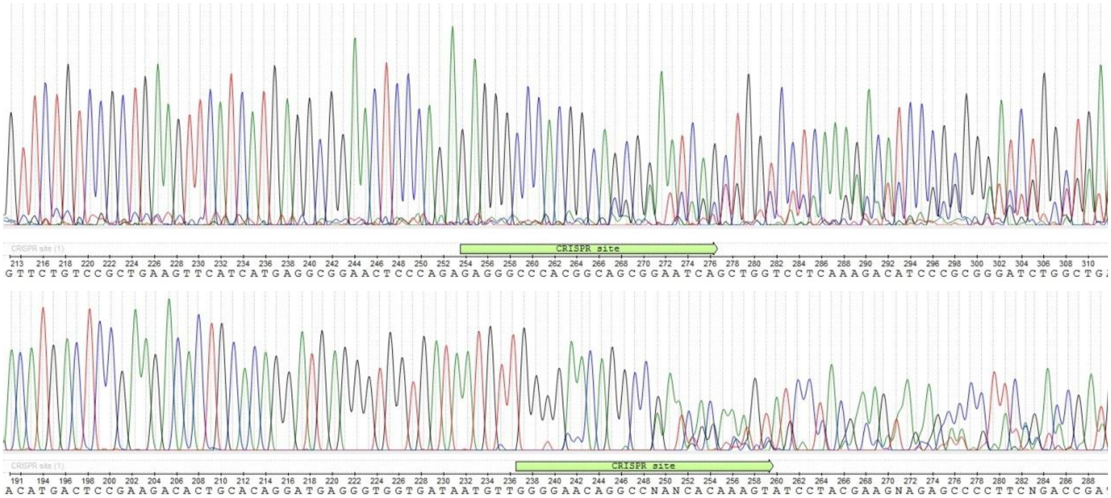

C *dnd/alb* KO fish, female

alb

dnd

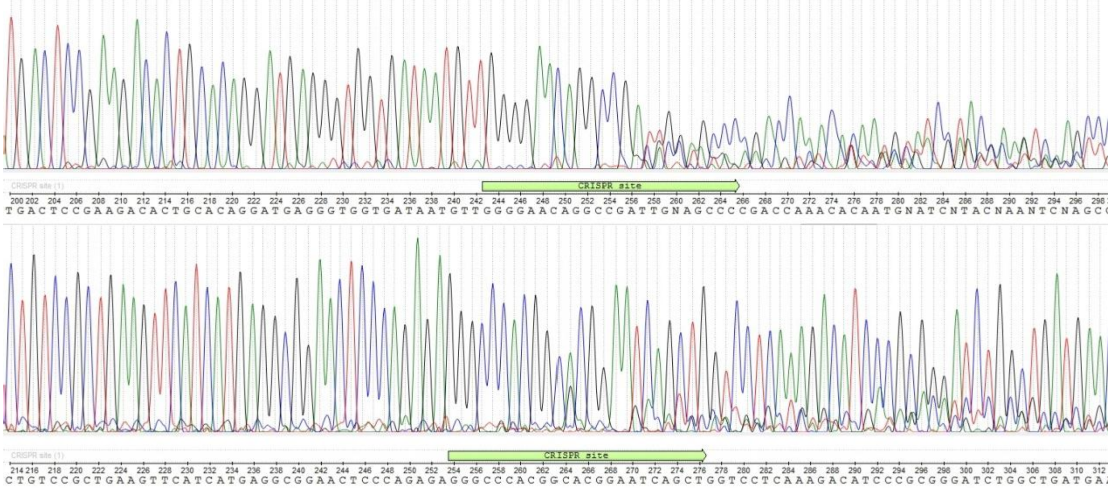

Supplementary Figure S2

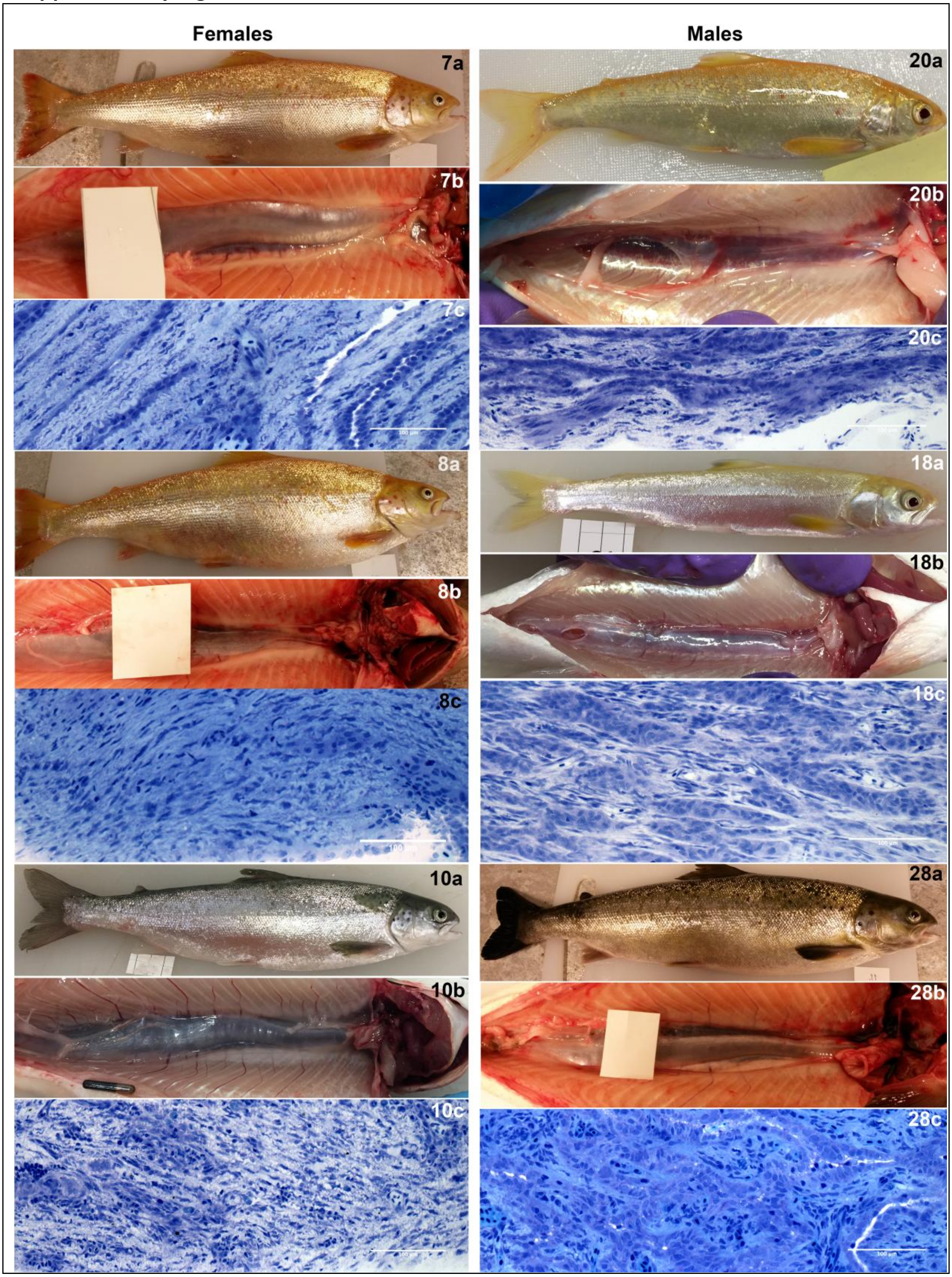

Supplementary Figure S3

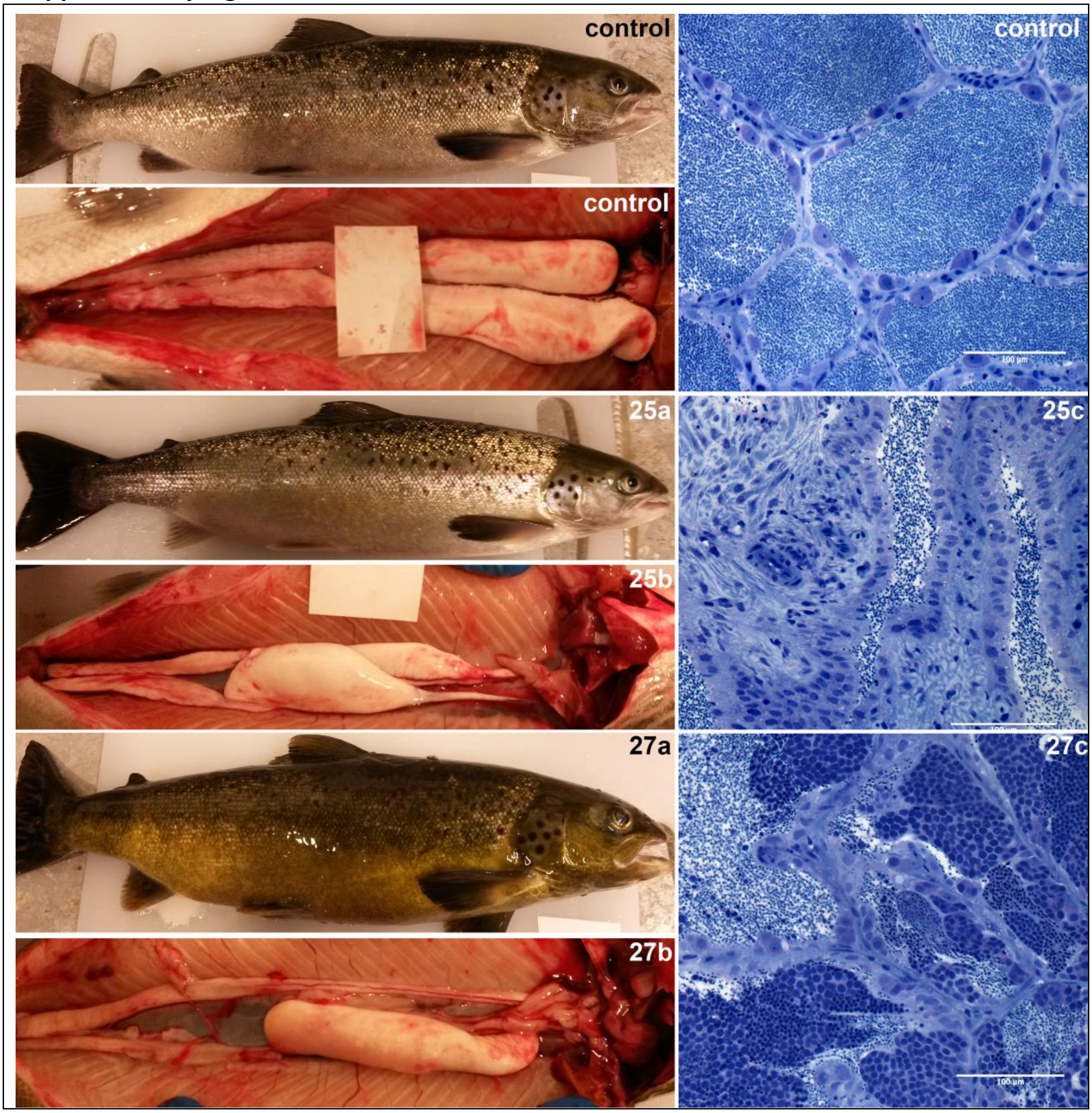

Supplementary Figure S4

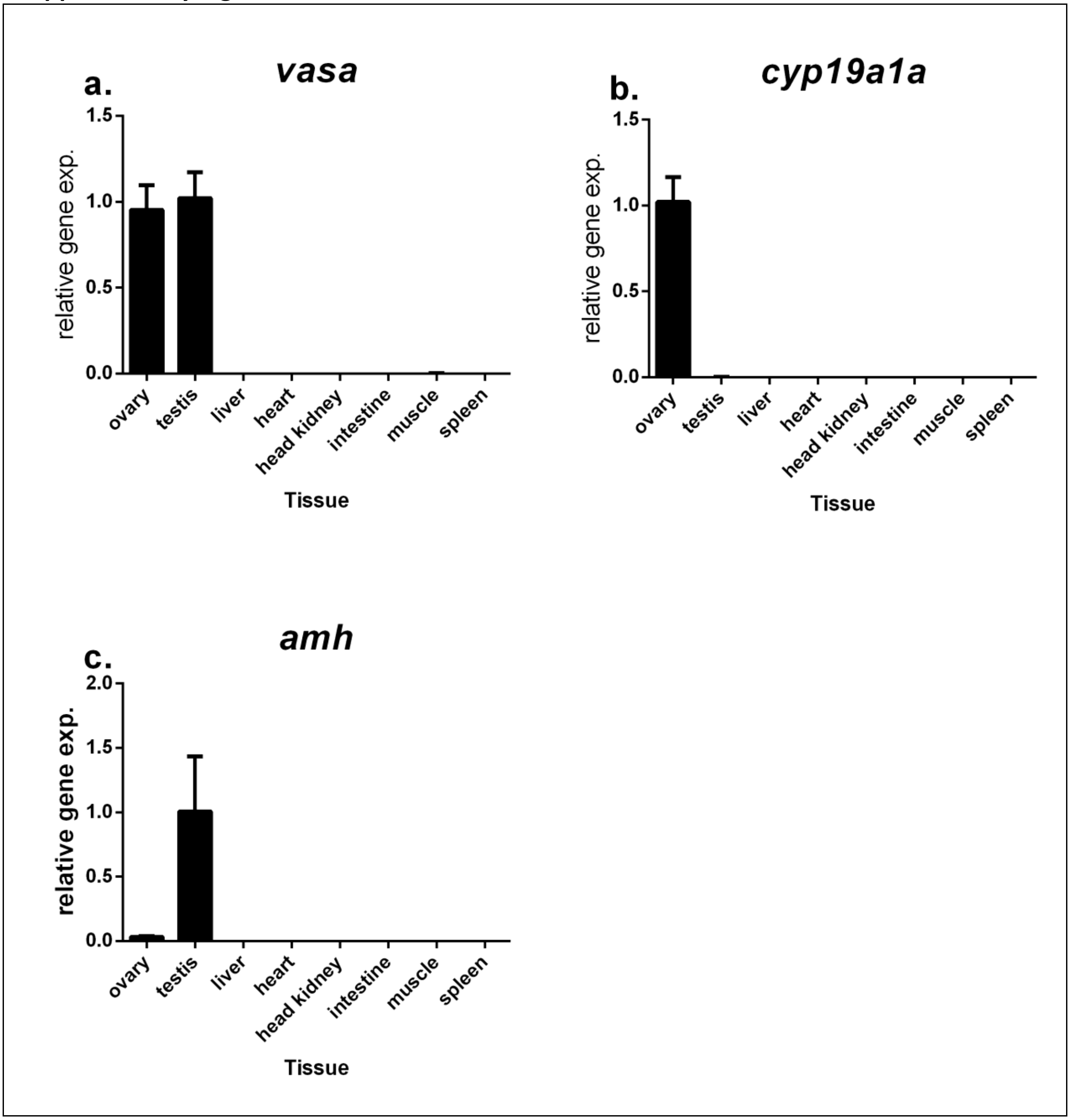

Supplementary Figure S5

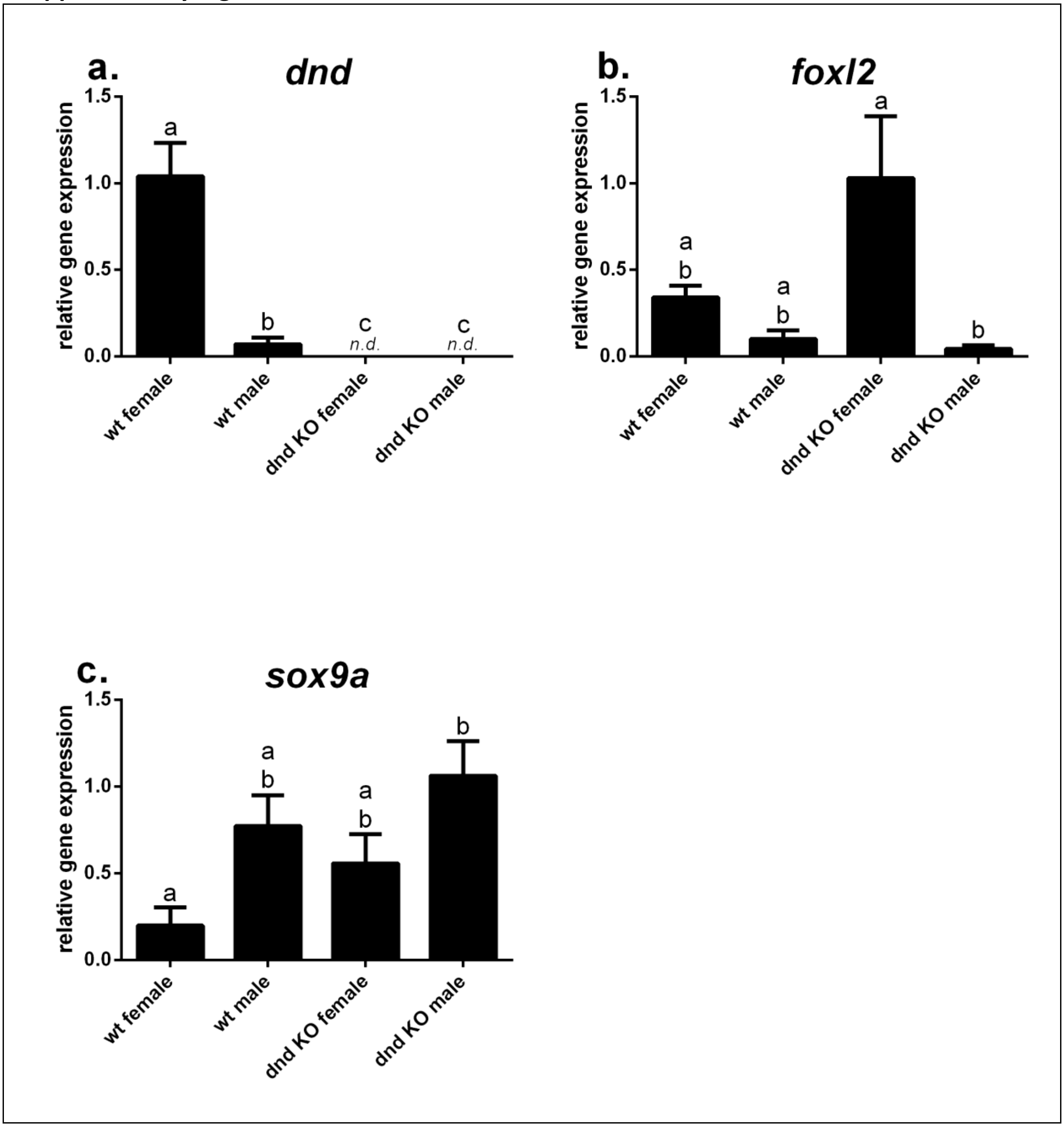

Supplementary table S1. Oligonucleotides and primers

CRISPR target site oligonucleotides

Oligo name	Oligo
dnd target	5’-GGGCCCACGGCACGGAACAGCGG-3’
dnd 1F	5’-TAGGGCCCACGGCACGGAACAG -3’
dnd 1R	5’-AAACCTGTTCCGTGCCGTGGGC-3’

PCR primers used in mutation screens for *dnd*

Primer name	Primer sequence
dnd PCRF	5’-TACATGCATCATTCCCACCCC-3’
dnd PCRR	5’-AAGTTCCACCATTACACTGCTT-3’

Primers and probes used for qPCR

Primer/ probe name	Primer sequence
vasa F	5’-GAGGTCCTCAAGACTACAACTTGTGT-3’
vasa R	5’-GCACATTGCAGCCCTTCAGT-3’
vasa probe	Fam-AGCACCGGTCACACCATTCGTGAG-TAMRA
cyp19a1a F	5’-TCAAACAGAACCCTGACGTAG-3’
cyp19a1a R	5’-GCTCCCTTTCACCTATAGCAGTGT-3’
cyp19a1a probe	Fam-ACAGCTCCTAGAAGAGAT-MGB
amh F	5’-CAGTCACTCTCTGCAGCCTTACAA-3’
amh R	5’-CAACATTGAATCTCCATTTCAGTTTAC-3’
amh probe	Fam-TTTGCCCTCGGGTTGCTTTCCTGTT-TAMRA
ef1a F	5’-CCCCTCCAGGACGTTTACAAA-3’
ef1a R	5’- CAGACGGCCCACAGGTACA-3’
ef1a probe	Fam- ATCGGTGGTATTGGAAC-MGB
dnd F	5’-TCTGTACAGGGCCTGATGGT-3’
dnd R	5’-TAAAACAAAGTAGGGGATCTGTG-3’
foxl2 F	5’-TACTGGGATCACGAGAGCAAAC-3’
foxl2 R	5’- ACAAGTGGCCCTCTGGTAAAC-3’
sox9a F	5’-TGCAGAACTCAGCAAAACCC-3’
sox9a R	5’-TTTTCTCCTTCTGGGCTGGTAC-3’

Supplementary table S2. Overview of all fish sampled and used in the analysis of data.

No	Sample	sex[1]	Phenotype	qPCR	Sampling date	Length	Weight	Age (months)
1[2]	Control	F	Normal immature female	Yes	20/1-2015	25,8	186	15
2	Control	F	Normal immature female	Yes	20/1-2015	29,9	309	15
3	Control	F	Normal immature female	Yes	20/1-2015	28,1	246	15
4[2]	*dnd/alb* KO	F	Female lacking ovarian bulbs.	No	20/1-2015	16	49	12
5	*dnd/alb* KO	F	Female lacking ovarian bulbs.	Yes	20/1-2015	18,9	62	12
6	*dnd/alb* KO	F	Female lacking ovarian bulbs.	No	12/8-2015	43	1063	18
7[3]	*dnd/alb* KO	F	Female lacking ovarian bulbs.	No	12/8-2015	42,5	1022	18
8[3]	*dnd/alb* KO	F	Female lacking ovarian bulbs.	No	12/8-2015	42	1051	18
9	*dnd/alb* KO	F	Female lacking ovarian bulbs.	No	12/8-2015	42,5	1072	18
10[3]	*dnd* KO	F	Female lacking ovarian bulbs.	Yes	20/1-2015	28,3	255	15
11	*dnd* KO	F	Female lacking ovarian bulbs.	Yes	20/1-2015	27	248	15
12	*dnd* KO	F	Female lacking ovarian bulbs.	Yes	20/1-2015	24	153	15
13	*dnd* KO	F	Normal immature female	No	20/1-2015	27	229	15
14	Control	M	Immature male	Yes	12/3-2015	26,5	208	17
15[2]	Control	M	Immature male	Yes	12/3-2015	20,5	95	17
16	Control	M	Immature male	Yes	12/3-2015	21,5	122	17
17	*dnd/alb* KO	M	Male lacking germ cells with tubular structure.	Yes	20/1-2015	20	77	12
18[3]	*dnd/alb* KO	M	Male lacking germ cells with tubular structure.	Yes	20/1-2015	17,3	40	12
19[2]	*dnd/alb* KO	M	Male lacking germ cells with tubular structure.	Yes	12/3-2015	13.5	31	14
20[3]	*dnd/alb* KO	M	Male lacking germ cells with tubular structure.	Yes	12/3-2015	22	110	14
21	*dnd* KO	M	Male lacking germ cells with tubular structure.	No	20/1-2015	26	191	15
22	*dnd* KO	M	Male lacking germ cells with tubular structure.	No	20/1-2015	27,5	231	15
23	*dnd KO*	M	Immature male,	No	20/1-2015	25,2	191	15
24	*dnd KO*	M	Maturing male with half gonad- mosaic	No	20/1-2015	27	254	12
25[4]	*dnd* KO	M	Maturing male with mosaic gonad	No	12/8-2015	34	548	20
26	*dnd* KO	M	Maturing male with mosaic gonad	No	12/8-2015	30	363	20
27[4]	*dnd KO*	M	Maturing male with mosaic gonad	No	12/8-2015	37	724	20
28[3]	*dnd KO*	M	Male lacking germ cells with tubular structure.	No	12/8-2015	41,5	1012	20
29	*dnd KO*	M	Male lacking germ cells with tubular structure.	No	12/8-2015	44	1322	20
30	*dnd KO*	M	Male lacking germ cells with tubular structure.	No	12/3-2015	38	781	20

[1] sdy PCR determined genetic sex
[2] Fish shown in Figure 1
[3] Fish shown in Supplementary figure S2
[4] Fish shown in Supplementary figure S3
